# The Child and Youth Mental Health Assessment (ChYMH): An examination of the psychometric properties of an integrated assessment developed for clinically referred children and youth

**DOI:** 10.1186/s12913-016-1970-9

**Published:** 2017-01-26

**Authors:** Shannon L. Stewart, Chloe A. Hamza

**Affiliations:** 1 Faculty of Education, Western University, 1137 Western Road, London, ON N6G 1G7 Canada; 2 University of Toronto, Ontario Institute for Studies in Education, 252 Bloor Street West, Toronto, ON M5S 1V6 Canada

**Keywords:** interRAI, Mental health, Children, Assessment

## Abstract

**Background:**

The Child and Youth Mental Health (ChYMH) assessment system was developed by interRAI (i.e., an international collective of researchers and clinicians from over thirty countries) in response to the unprecedented need for a coordinated approach to delivery of children’s mental health care. Many interRAI instruments are used across Canada and internationally, but the ChYMH represents the first assessment specifically for children and youth. In the present paper, a short overview of the development process of the ChYMH is provided, and then the psychometric properties of several embedded scales on the ChYMH are examined.

**Methods:**

Participants included 1297 children and youth and their families who completed the ChYMH after being referred to mental health agencies within Ontario, Canada. In addition, smaller subsets of participants (*N* = 48–53) completed additional criterion measures, including the Social Skills Improvement System (SSIS), the Child and Adolescent Functional Assessment Scale (CAFAS), the Child Behavior Checklist (CBCL), and the Brief Child and Family Phone Interview (BCFPI).

**Results:**

Results demonstrated that the ChYMH subscales had strong internal-consistency (Cronbach’s higher than .70), and correlated well with the criterion measures.

**Conclusions:**

Findings support the clinical utility of the ChYMH for use among clinically referred children and youth. Implications for children’s mental health assessment and practice are discussed.

## Background

In North America, one out of every five children experiences a mental health concern, such as depression, anxiety, attention-deficit hyperactivity disorder, or conduct problems [1, 2]. The stability, persistence, and aversive long-term outcomes of childhood illness are evident across the lifespan [3]. Over 50–75% of adult mental health issues have their onset in childhood or adolescence [4], and children who experience mental health issues are at increased risk for academic underachievement [5, 6], underemployment [7], criminal activity [8, 9], and risk for suicide [10]. Given the compounding effects of mental health issues over time, as well as the staggering economic burden of mental health costs in North America [1, 11], ensuring children have access to timely, responsive, and integrated services is critically important; however, the children’s mental health care system is underfunded and fragmented, often referred to as the “orphan’s orphan” of health care [12]. Indeed, over 75% of children who experience mental health issues, do not receive access to appropriate treatment [13].

For over the past two decades, the systems-of-care philosophy in mental health has recognized the need to respond to a fragmented service system through greater integration and coordination [14]. In response to the need for a coordinated approach to the delivery of mental health care in Canada, there have been significant advances within the adult mental health sector. More specifically, the call to develop an effective information system that provides a comprehensive, scientifically rigorous approach to mental health assessments led to the Resident Assessment Instrument-Mental Health (RAI-MH). This instrument was developed by interRAI (i.e., an international not-for-profit collective of researchers and clinicians from over thirty countries committed to improving the care of vulnerable persons through a seamless approach to assessment across a variety of service sectors) in collaboration with the Ontario Joint Policy and Planning Committee [15]. In 2006, the Ontario Ministry of Health and Long-Term Care mandated the use of the interRAI RAI-MH, given its ability to provide a comprehensive assessment of adult mental health needs, and support evidenced-based care planning across service settings. Moreover, the assessment has been used for the purposes of outcome measurement, quality improvement, and case-mix, and compliments interRAI’s existing suite of instruments for use across a variety of service sectors (e.g., home care, acute care, long-term care, etc.).

Despite the advances in adult mental health care in Canada, the children’s mental health care system has continued to lack a comprehensive and psychometrically sound tool for integrated use across a variety of service sectors (e.g., inpatient/outpatient mental health, hospitals, youth justice sites, schools, etc.). Although numerous assessments have been developed to assess child functioning, these assessments are often narrowly focused (e.g., assesses symptoms for a given mental health problem, such as depression). As a result, multiple assessments are often required to obtain a comprehensive understanding of the child/youth’s mental health needs, resulting in redundancies in information collected, additional clinical time and resources, as well as increased assessor burden on children and their families [16, 17]. Moreover, previous assessments have yet to facilitate an integrated and coordinated approach to children’s mental health across various service sectors, or facilitate a lifespan approach to the delivery of mental health care (i.e., from childhood through to late adulthood). Given the strong need for a coordinated approach, the interRAI Child and Youth Mental Health assessment (ChYMH) was developed to facilitate a comprehensive, standardized, and integrated approach to the delivery of mental health services in Canada to further support a life course approach to assessment.

The development of the ChYMH was a rigorous and arduous process that included the involvement of over 100 clinical experts from nine over countries (e.g., Canada, United States, Poland, Belgium, Finland, Sweden, Netherlands, Australia and Czech Republic). The development process included several stages, to ensure a peer-reviewed high quality end product with the potential for widespread uptake not only in Canada, but around the globe. First, a core research team in Canada conducted extensive literature reviews on all available existing children and youth mental health assessments, the etiology of mental health issues among children and youth ages 4–18 years of age, as well as the best-practice guidelines for children’s mental health care. As part of this literature review, domains requiring assessment were identified (e.g., mental health, physical health, cognitive functioning, strengths and resilience, stress and trauma, etc.), and relevant items pertinent to children and youth were adapted from other interRAI instruments (e.g., RAI-MH). After the literature review was conducted, panels of expert working groups were established (including clinicians, researchers, front line mental health workers, policy makers, etc.). These groups specifically focused on ChYMH item creation to ensure that identified domains were well-represented, items were clinically useful and relevant, and that the instrument had strong face validity. Once items were finalized by these working groups, the items were then presented to two international groups, the interRAI Network of Excellence in Mental Health (iNMH) and the interRAI Instrument and Survey Development Committee (ISD). These committees collectively included over 40 internationally renowned experts in mental health, survey development, measurement and content. The expert committees provided feedback on each item, which was then incorporated by the working groups into the ChYMH. The built-in collaborative action plans (which provide real-time evidence-informed recommendations for care planning around areas of risk), were developed through a similar process.

The interRAI Child and Youth Mental Health assessment has had widespread uptake in Ontario as a standard of care at over 60 mental health agencies in the province. Moreover, the ChYMH has led to the development of complimentary child and youth assessments [e.g., the Child and Youth Mental Health Assessment for youth with developmental disabilities (ChYMH-DD)] that are designed to act in combination as an integrated children’s mental health suite. Despite the widespread uptake of the ChYMH, however, published research on the psychometric properties of the instrument is lacking. In contrast, there have been numerous reliability and validity studies conducted across the family of interRAI instruments for adults. Indeed, the adult interRAI assessments have been shown to demonstrate good psychometric properties, including strong inter-rater reliability, convergent validity, and inter-item reliability [15, 18–21]. Although the ChYMH was adapted from the existing interRAI assessments where possible, additional research is still needed to specifically examine the psychometric properties of the ChYMH among clinically referred children and youth.

### Present study

Although the ChYMH has already had widespread uptake in Ontario because of its strong clinical utility and multiple applications (e.g., care planning, program evaluation, case-mix), research on the psychometric properties of the instrument has yet to be published. In the present study, we provide an empirical investigation of the psychometric properties of the ChYMH. More specifically, data collected from several mental health agencies in Ontario was used to assess the inter-item reliability of several embedded scales on the ChYMH (i.e., Aggressive/Disruptive Behavior Scale Anhedonia Rating Scale, Anxiety Scale, Caregiver Distress Scale, Communication Scale, Cognitive Functioning Scale, Depressive Symptoms Scale, Distractibility/Hyperactivity Scale, Peer Conflict Scale, Sleep Difficulties Scale). In addition, participants of the larger sample agreed to complete additional existing child and youth mental health measures with relevant domains, to assess scale criterion validity.

## Method

### Participants

The present sample was comprised of 1297 children and youth (65% male) between the ages of 4–18 years (*M*age = 11.2, SD = 3.45) who completed the ChYMH as part of normal clinical practice across 15 sites providing mental health services to children and youth within the province of Ontario. Given that the assessment was completed as a standard of care, there was a very high response rate of completion (over 90%). At the time of referral to clinical care, 91% of children and youth lived with their parents or primary guardian, 1% lived alone, 2% lived with other relatives, 4% lived with a foster family and 2% lived with a nonrelative (but not a foster family). Among those children and youth referred for assessment at time of intake into care, 22% had no contact with a community mental health agency or professional with the past year, 28% had contact within 31 days or more, and 50% had contact within the last 30 days.

### Measures - ChYMH

The interRAI ChYMH includes over 400 items, and builds a comprehensive picture of the child/youth’s strengths, needs, functioning and areas of risk to inform care planning for clients with mental health needs [22]. The interRAI ChYMH is based on a semi-structured interview format, and trained assessors complete the instrument using all sources of information, including direct contact with the family and their child or youth, as well as other service providers and records (e.g., teachers, clinical charts and observations). In the present study, the internal reliability and criterion validity of several embedded scales assessing children and youth’s mental health and family functioning were assessed (see Table 1 for a list of domains).

**Table 1:** Means on ChYMH scales

	Means-based comparison by sex		Means-based comparison by age		
Scale	Mean (Males)	Mean (Females)	Mean (4–12 years)	Mean (13–18 years)	Grand Mean
Aggressive/Disruptive behavior Scale	6.99(5.15)^a^	5.23(4.87)^b^	7.13(5.29)^a^	5.09(4.54)^b^	6.37 (5.12)
Anhedonia Rating Scale	3.44(4.05)^a^	3.60(4.04)^a^	2.83(3.65)^a^	4.66(4.34)^b^	3.50 (4.05)
Anxiety Scale	6.60(5.51)^a^	7.15(5.77)^a^	7.12(5.80)^a^	6.78(5.27)^a^	6.99 (5.61)
Caregiver Distress Scale	0.76(0.82)^a^	0.65(0.76)^b^	0.66(0.79)^a^	0.83(0.83)^b^	0.72 (0.80)
Communication Scale	1.40(1.61)^a^	0.93(1.34)^b^	1.32(1.61)^a^	1.07(1.39)^b^	1.23 (1.54)
Cognitive Functioning Scale	2.38(1.60)^a^	1.70(1.60)^b^	2.30(1.62)^a^	1.89(1.60)^b^	2.14 (1.63)
Depressive Symptoms Scale	11.38(7.12)^a^	12.64(7.77)^b^	11.45(7.35)^a^	12.47(7.49)^b^	11.82 (7.41)
Distractibility/Hyperactivity Scale	6.90(5.51)^a^	7.15(5.78)^b^	9.89(5.00)^a^	7.94(4.63)^b^	9.17 (4.95)
Peer Conflict Scale	0.43(0.81)^a^	0.43(0.77)^a^	0.39(0.76)^a^	0.52(0.86)^b^	0.43 (0.80)
Sleep Difficulties Scale	4.20(3.81)^a^	4.40(3.97) ^a^	4.17(3.79)^a^	4.46(4.01)^a^	4.27 (3.87)

*N* = 1297, higher scores indicate greater risk. Within the means-based comparison by sex column, different superscripts equal significant differences between males and females at *p* < 0.05. Within the mean-based comparison by age column, different superscripts equal significant differences between younger and older children and adolescents at *p* < 0.05

#### Aggressive/Disruptive Behavior Scale (ADBS)

The 5-item ADBS scale assessed the frequency and severity of aggressive and disruptive behavior (i.e., physical abuse, verbal abuse, socially inappropriate or disruptive behavior, destructive behavior toward property, outbursts of anger). The frequency of each behavior was assessed using a 4-point scale (0 = *not present* to 4 = *Exhibited daily in last 3 days, 3 or more episodes or continuously*), which was totaled to provide a composite score (from 0 to 20). Higher scores indicated higher levels of aggressive/disruptive behavior.

#### Anhedonia Rating Scale

The 4-item Anhedonia Rating Scale for children and youth assessed frequency of symptoms of anhedonia (i.e., lack of interest in social interaction, lack of motivation, anhedonia, and withdrawal from activities of interest). The frequency of each symptom was assessed using a 4-point scale (0 = *not present* to 4 = *Exhibited daily in last 3 days, 3 or more episodes or continuously*), which was totaled to provide a composite score (from 0 to 16). Higher scores indicate higher levels of anhedonia.

#### Anxiety Scale

The seven-item Anxiety Scale assessed the frequency of several symptoms of anxiety (i.e., repetitive anxious concerns, unrealistic fears, obsessive thoughts, compulsive behavior, intrusive thoughts or flashbacks, episodes of panic, nightmares). The frequency of each symptom was assessed using a 4-point scale (0 = *not present* to 4 = *Exhibited daily in last 3 days, 3 or more episodes or continuously*), which was totaled to provide a composite score (from 0 to 28). Higher scores indicate higher levels of anxiety.

#### Caregiver Distress Scale

The Caregiver Distress Scale assessed the presence and diversity of three significant caregiver well-being factors, including whether the parent/primary guardian had experienced major life stressors in the last 90 days, was unable or unwilling to continue in caring activities, or expressed feelings of distress, anger or depression. A total score of 0–3 was given, with higher scores indicating greater caregiver distress.

#### Communication Scale

The child/youth’s ability to communicate was assessed with two items: 1) expression (i.e., child’s ability to make self-understood), and 2) comprehension (i.e., the child’s ability to understand others). Expression was assessed on a 4-point scale from (0 = *understood* to 4 = *rarely or never understood*) and comprehension was scored on a 4-point scale from (0 = *understands* to 4 = *rarely or never understands*). A total score of 0–8 is given, with higher scores indicating greater difficulties with communication.

#### Cognitive Functioning Scale

Cognitive functioning was assessed using five items, including cognitive skills for decision making (0 = *Independent* to 5 = *No discernible consciousness*), short-term memory (0 = *no memory problem*, 1 = *memory problem present*), procedural memory (0 = *no memory problem*, 1 = *memory problem present*), making self-understood (0 = *understood* to 4 = *rarely or never understood*), and ability to understand others (0 = *understands* to 4 = *rarely or never understands*). For the purposes of the study, the cognitive functioning scale was collapsed into an interval scale from 0 to 5 (with the presence of each cognitive impairment coded as a 1) to evaluate inter-item reliability and criterion validity.

#### Depressive Symptoms Scale

The 9-item Depressive Symptoms Scale assessed the frequency of depressive symptoms (e.g., crying/tearfulness, expressions of hopelessness, irritability, sad, pained or worried facial expressions). The frequency of each symptom was assessed using a 4-point scale (0 = *not present* to 4 = *Exhibited daily in last 3 days, 3 or more episodes or continuously*), which was totaled to provide a composite score (from 0 to 36). Higher scores indicated higher levels of depressive symptoms.

#### Distractibility/Hyperactivity Scale

The 4-item Distractibility/Hyperactivity Scale assessed the frequency of four facets of distractibility and hyperactivity (i.e., impulsivity, ease of distraction, hyperactivity and disorganization). The frequency of each behavior was assessed using a 4-point scale (0 = *not present* to 4 = *Exhibited daily in last 3 days, 3 or more episodes or continuously*), which was totaled to provide a composite score (from 0 to 16). Higher scores indicated higher levels of distractibility and hyperactivity.

#### Peer Conflict Scale

The 3-item Peer Conflict Scale assessed the presence of peer difficulties (i.e., conflict with or repeated criticism of close friends, friends are persistently hostile or critical of child/youth, pervasive conflict with peers (exclude close friends). A total score of 0–3 was given, with higher scores indicating greater peer conflict.

#### Sleep Difficulties Scale

The 4-item Sleep Difficulties Scale assessed the frequency of four sleep problems [i.e., difficulty falling asleep or staying asleep, wakes multiple times a night, falls asleep during the day (excluding naptime), resists bedtime]. The frequency of each sleep problem was assessed using a 4-point scale (0 = *not present* to 4 = *Exhibited daily in last 3 days, 3 or more episodes or continuously*), which was totaled to provide a composite score (from 0 to 16). Higher scores indicated higher levels of sleep difficulties.

### Measures - criterion measures

#### Social Skills Improvement System (SSIS)

In the present study, 53 caregivers completed the SSIS that assessed the child/youth’s social skills and problem behaviors [23]. The SSIS, which is written at a fifth grade reading level, required parents to indicate the frequency of 79 behaviors (e.g., acts sad or depressed, fights with others) on a 4-scale (1 = *never* to 4 = *almost always*). Using parents’ responses, scores on 10 subscales were derived, including externalizing behaviors, internalizing behaviors, communication, cooperation, assertion, responsibility, empathy, engagement, self-control, bullying, and hyperactivity/inattention. The SSIS has been shown to have good internal consistency, test-retest reliability, and strong criterion validity [23, 24].

#### Child and Adolescent Functional Assessment Scale (CAFAS)

Clinicians completed the CAFAS for 48 children and youth to assess the child/youth's level of functioning in a variety of settings (e.g., at school, at home, in the community) [25]. Clinicians rated the child/youth’s level of impairment on a scale from 0 = *minimal or no impairment* to 30 = *severe impairment* across eight primary domains: school, home, community, behavior towards others, moods/emotion, self-harm, substance use, and thinking. Assessors also evaluated the extent to which the caregiver struggled to provide support for the child (i.e., caregiver material needs), as well as the level of family social support (i.e., caregiver support). The CAFAS has been shown to have good inter-reliability, as well as strong criterion validity [26, 27].

#### Child Behavior Checklist (CBCL)

For the present study, the CBCL was completed by parents of 47 children and youth [28]. The CBCL requires parents to indicate the extent to which the child experiences several problem behaviors on a 3-point scale (0 = *not true* to 2 = *very true or often true*). The CBCL includes eight subscales assessing several domains: anxious/depressed, withdrawn/depressed, somatic complaints, social problems, thoughts problems, attention problems, rule breaking behavior and aggressive behavior. We also included an assessment of school total, which is a composite measure of school performance, school problems, grade repetition, and whether special education is required (i.e., higher scores, better school outcomes). The CBCL has been shown to have strong reliability and validity in previous research [29].

#### Brief Child and Family Phone Interview (BCFPI)

A standardized structured interview [30], typically administered by phone, was conducted with parents of 45 children and youth. Assessors asked parents several questions about the child to assess child functioning using nine subscales, including: 1) regulating attention; 2) regulating impulsivity and activity level; 3) regulating attention, impulsivity and activity level; 4) cooperativeness; 5) conduct; 6) separating from parents; 7) managing anxiety; 8) managing mood and 9) managing mood and self-harm. The BCFPI has been shown to have strong test-retest reliability, and validity in previous research [31, 32].

### Procedure

Trained assessors collected data at time of the child/youth’s intake into clinical care at one of the 15 mental health service providers in Ontario, Canada. Each assessor involved in the study had at least two years of clinical experience with children and youth, and also completed a comprehensive training program on the administration of the interRAI ChYMH. Each assessment took approximately 60–90 min to complete, and as part of the assessment process, assessors gathered information from the child/youth, the primary caregivers, teachers and used any additional clinical data (e.g., medical or educational files). The study was approved by the University of Western Ontario ethics board (REB #106415).

### Plan of analysis

First, scale means were calculated, and age and sex differences on scale means were examined. Next, inter-item reliability was examined by assessing the inter-item correlations of the scales using the full sample (N =1297). Items on scales were not only summed, but each item assessed was similarly rated and equally contributed to the total score [33]. Second, criterion validity was assessed within the subsamples of participants who completed additional assessments (*N* =47–53) by examining the correlations between the scales on the ChYMH and the criterion measures.

## Results

### Preliminary results

The means on each of the 10 scales (i.e., Aggressive/Disruptive Behavior Scale, Anhedonia Rating Scale, Anxiety Scale, Caregiver Distress Scale, Communication Scale, Cognitive Functioning Scale, Depressive Symptoms Scale, Distractibility/Hyperactivity Scale, Peer Conflict Scale, Sleep Difficulties Scale) are presented for the entire sample (as well as by sex and age) in Table 1. As compared to males, females were at lower risk on the Aggressive/Disruptive Behavior Scale, *t*(1295) = 5.957, *p* < 0.001, the Communication Scale *t*(1088.76) = 5.529, *p* < 0.001, the Cognitive Functioning Scale *t*(1295) = 7.284, *p* < 0.001, and the Distractibility/Hyperactivity Scale, *t*(882.52) = 6.547, *p* < 0.001. Females were at higher risk than males on the Caregiver Distress Scale, *t*(1260) = 2.218, *p* < 0.05, and the Depressive Symptoms Scale, *t*(1295) = −2.924, *p* < 0.01.

Compared to children (ages 4–12), youth (ages 13–18) were at lower risk on the Aggressive/Disruptive Behavior Scale, *t*(1290) = 7.302, *p* < 0.001, Communication Scale, *t*(1104.45) = 3.026, *p* < 0.01, Cognitive Functioning Scale, *t*(1290) = 4.357, *p* < 0.001, and the Distractibility/Hyperactivity Scale, *t*(1290) = 4.357, *p* < 0.001. Youth were at higher risk on the Anhedonia Rating Scale, *t*(841.87) = −7.626, *p* < 0.001, the Caregiver Distress Scale, *t*(1255) = −3.543, *p* < 0.001, the Depressive Symptoms Scale *t*(1290) = −2.389, *p* < 0.05, and the Peer Conflict Scale, *t*(806.57) = −2.557, *p* < 0.05, compared to children.

### Primary results

Inter-item correlations are presented in Table 2 for the entire sample. Overall, the scales demonstrated strong inter-item reliability (i.e., Cronbach’s alpha of .70 or greater for all scales, except for the Sleep Scale which was 0.66). The correlations between the ChYMH scales and the corresponding criterion measures are presented in Table 3. As predicted, the ChYMH subscales were correlated with relevant criterion measures. The strongest correlations were between the ChYMH Depressive Symptoms Scale and the criterion scales of the CBCL anxious/depressed scale (*r* = .61), BCFPI Managing mood and self-harm scale (*r* = .61), and the SSIS Internalizing behavior scale (*r* = .60). In addition, there were strong correlations between the ChYMH Anhedonia Rating Scale and the managing mood and self-harm scale on the BCFPI (*r* = .60), as well as the ChYMH Peer Conflict Scale and criterion scales of the CBCL social problems scale (*r* = .60), and the SSIS externalizing subscale (*r* = .57). The Aggressive/Disruptive Behavior Scale on the ChYMH was also strongly correlated with the SSIS Self-Control Subscale (*r* = −.58).

**Table 2:** Internal consistency of ChYMH subscales

Scale	Inter-item consistency
Aggressive/Disruptive Behavior Scale	0.83
Anhedonia Rating Scale	0.73
Anxiety Scale	0.71
Caregiver Distress Scale	0.73
Communication Scale	0.76
Cognitive Functioning Scale	0.70
Depressive Symptoms Scale	0.80
Distractibility/Hyperactivity Scale	0.78
Peer Conflict Scale	0.88
Sleep Difficulties Scale	0.67

*Note: N* = 1297, Cronbach’s alphas are presented. Given that there were only two items on the Communication Scale, the Spearman Brown Coefficient (split-half) is provided

**Table 3:** Criterion validity – correlations between ChYMH scales and criterion measures

ChYMH scale	Criterion scale	*r*
Aggressive/Disruptive Behavior Scale	SSIS: Externalizing behaviors SSIS: Cooperation SSIS: Responsibility SSIS: Self-control SSIS: Bully behavior CAFAS: Behavior toward others CBCL: Aggressive behaviors CPCL: Social problems BCFPI: Cooperation BCFPI: Externalizing behaviors	.52*** -.45** -.49*** -.58*** .42** .50*** .50*** .42** -.52*** .42**
Anhedonia Rating Scale	SSIS: Internalizing behaviors CBCL: Withdrawal BCFPI: Managing mood BCFPI: Managing mood and self-harm BCFPI: Internalizing behaviors BCFPI: Social participation	.44** .53*** .56*** .60*** .35** .51***
Anxiety Scale	SSIS: Internalizing behaviors SSIS: Self-control SSIS: Cooperation SSIS: Hyperactivity-inattention CBCL: Anxious/depressed CBCL: Social problems CBCL: Thought problems CBCL: Aggressive behaviors	.43** -.44** -.35** .49*** .42** .42** .42** .42**
Caregiver Distress Scale	SSIS: Bully SSIS: Self-control CAFAS: Caregiver material needs CBCL: Anxious/depressed CBCL: Social problems	.32* -.29* .31* .38** .31*
Communication Scale	CBCL: Social problems	.33*
Cognitive Functioning Scale	CAFAS: Home problems CBCL: Social problems CBCL: School total	.36** .28* -.39**
Depressive Symptoms Scale	SSIS: Internalizing behaviors SSIS: Self-control SSIS: Hyperactivity/inattention CAFAS: Self-harm CBCL: Anxious/depressed BCFPI: Managing mood BCFPI: Managing mood and self-harm BCFPI: Internalizing behaviors	.60*** -.37** .39** .38** .61*** .53*** .60*** .48**
Distractibility/Hyperactivity Scale	SSIS: Self-control SSIS: Hyperactivity/inattention BCFPI: Regulating attention BCFPI: Regulating impulsivity and activity BCFPI: Regulating attention, impulsivity and activity BCFPI: Externalizing	-.37** .44** .38** .51*** .53*** .44**
Peer Conflict Scale	SSIS: Externalizing SSIS: Responsibility SSIS: Self-control SSIS: Bullying SSIS: Hyperactivity/inattention CAFAS: School problems CAFAS: Caregiver support CBCL: Social problems CBCL: Rule-breaking CPCL: Aggressive behaviors BCFPI: Conduct	.57*** .52*** -.43** .51*** .45** .43** .50** .60*** .51*** .54*** .44**
Sleep Difficulties Scale	SSIS: Assertiveness CBCL: Somatic complaints	-.32* .41*

Note: *SSIS* Social Skills Improvement System, *CAFAS* Child and Adolescent Functional Assessment, *CBCL* Child Behavior Checklist, *BCFPI* Brief Child and Family Phone Interview. Significant correlations are provided at *** = *p* < 0.001, ** = *p* < 0.01, * = *p* < 0.05

## Discussion

The ChYMH was developed by interRAI in collaboration with leading experts in children’s mental health, to facilitate a comprehensive, standardized and integrated (cross-sector) approach to the assessment of children’s mental health needs. Moreover, the ChYMH was designed to foster continuity in assessment across the lifespan (from early childhood through to late adulthood) within the interRAI suite of instruments. Despite the widespread uptake of the ChYMH in Canada, there is a lack of published research on the psychometric properties of the instrument. To address this important limitation, in the present study the inter-item reliability and criterion validity of several scales on the ChYMH were evaluated using data collected from 15 mental health agencies in Ontario. It was found that the embedded scales on the ChYMH (i.e., Aggressive/Disruptive Behavior Scale, Anhedonia Rating Scale, Anxiety Scale, Caregiver Distress Scale, Communication Scale, Cognitive Functioning Scale, Depressive Symptoms Scale, Distractibility/Hyperactivity Scale, Peer Conflict Scale, Sleep Difficulties Scale) demonstrated strong inter-item reliability, and correlated well with several criterion measures. Findings support the clinical utility of the ChYMH for use among clinically referred children and youth.

The findings of the present study are consistent with previous research demonstrating that interRAI adult assessment instruments have strong psychometric properties, including inter-item consistency and criterion validity [15, 18–21]. All of the embedded scales assessed on the ChYMH demonstrated strong inter-item reliability, which suggests that the extensive construction process of the ChYMH yielded scales with items assessing conceptually similar domains well. Further, ChYMH scales were found to correlate with clinically similar domains on existing psychological assessments (e.g., Child Behavior Checklist, Brief Child and Family Phone Interview). In particular, ChYMH scales assessing internalizing behaviors (e.g., depression, anxiety) and externalizing behaviors (e.g., aggressive symptoms) showed strong correlations with other problem behavior assessment scales. Given that both internalizing and externalizing behaviors are widely occurring mental health concerns among clinically referred children and youth, the ChYMH can provide a comprehensive assessment of these problem areas [4, 34].

Also noteworthy, are our findings around sex and age differences on the scales. More specifically, it was found that female children and youth were at higher risk for depressive symptoms and caregiver distress, whereas males were at higher risk for aggressive/disruptive behavior, distractibility/hyperactivity, communication problems and cognitive functioning difficulties. Our findings are consistent with a larger body of research, which has found that males tend to present more with externalizing behaviors, whereas females tend to report higher levels of internalizing behaviors [35–37]. Moreover, our findings suggest that males may be more likely to be referred for clinical care than females, due to issues related to cognitive difficulties, problems with executive functioning (e.g., attention, memory) and communication issues (e.g., ability to speak with others), which have found to be associated with distractibility/hyperactivity in previous research [38, 39].

It also was found that children (ages 4–12) reported more aggressive/disruptive behavior, distractibility/hyperactivity, as well as communication and cognitive functioning issues relative to youth (ages 13–18). Given that externalizing behaviors tend to have their onset in early childhood, it is not surprising that children, compared to youth, reported higher levels of risk for aggressive, disruptive, and hyperactive behavior at time of intake [35, 40]. Moreover, attention problems may decline as children age, and regulatory capabilities strengthen with further brain development [41, 42]. In contrast, compared to children, youth were at greater risk for depressive symptoms, and anhedonia – which coincides with later onset of depressive symptomology in adolescence [43]. Research has shown that individuals lacking strong interpersonal relationships are at heightened risk for depressive symptoms, which is also consistent with findings that youth reported higher levels of peer conflict [44], compared to children.

Overall, the findings of the present study offer further support that the ChYMH is a rigorously developed comprehensive assessment which can serve to support the early identification of mental health concerns among children and youth, as well as facilitate evidence-informed care planning, program evaluation, and outcome measurement. Indeed, although the present study utilized data collected from children and youth at time of intake into clinical treatment, the ChYMH is designed for re-assessment specifically to monitor change over time among children and youth. The ChYMH may be preferable to other assessments that are often narrowly focused by providing a more comprehensive assessment. Moreover, the ChYMH can also work in combination with other interRAI instruments to provide clinical information about change and stability in mental health over time among persons accessing care across the lifespan.

In working toward developing a coordinated approach to the assessment and delivery of children’s mental health care, the ChYMH can be used at both inpatient and outpatient mental health agencies to identify emerging or existing mental health concerns among children and youth, and support evidenced-informed and consistent responses to children’s mental health concerns. Other interRAI Child and Youth instruments have been developed and adapted for use in other service sectors (e.g., emergency rooms, schools) to create a seamless approach to assessment, prioritization, and triaging to support referrals from one service sector to another. This integrated approach also supports waitlist reduction strategies, provides tailored assessment modules for individualized care and facilitates information sharing among service providers, hospitals, and agencies, while preventing assessment burden and duplication [45]. Importantly, the ChYMH was designed to complement the existing suite of interRAI assessments to allow for meaningful comparisons across service sectors and across the life course. Moving forward, data collected with the ChYMH could be linked longitudinally to examine developmental trajectories related to mental and physical health later in life, as well as to identify early predictors of more life course persistent symptomology and potential mitigating factors fostering resilience (e.g., effective early supports for families). In addition to contributing to continuity of care and assisting with transitions across service sectors, it will eventually allow opportunities to study rare diseases and disorders across the lifespan.

Despite the many strengths of the present study, including the use of a comprehensive mental health assessment designed specifically for children and youth, as well as a large clinical sample, the present study is not without limitations. First, the present sample consisted of children and youth referred for clinical care in the province of Ontario. Although the sample was large, and representative of the population of children and youth receiving clinical care in Ontario, additional studies assessing the use of the ChYMH in other clinical populations in varied geographic regions would increase confidence around the generalizability of the findings. In addition, despite the strong response rate among participants (given that the assessment was a standard of care), we could not compare youth (and their families) who refused to complete the assessment to youth who had an assessment completed. It is possible that families who agreed to the assessment differ relative to families who did not, and our results may not be generalizable to all families receiving care in Ontario. In addition, analyses for the criterion validity of the scales were conducted using smaller subsets of the large sample, resulting in less statistical power. Although having a larger subsample of participants complete the criterion measures may have resulted in stronger associations between the ChYMH scales and the criterion measures, conducting such studies are extremely challenging. Given the overwhelming demands of the children’s mental health care system in Canada, every effort is made to ensure that assessor burden on clinical staff, and the families of children receiving care, is limited. Thus, it is difficult to conduct additional assessments at time of intake into treatment (this limitation has also been noted by other researchers conducting comprehensive child and youth assessment) [46]. Nevertheless, our study provides the first published empirical investigation of the psychometric properties of the ChYMH, and is especially timely given its widespread uptake across the province of Ontario (i.e., the instrument is currently being supported for use in over 60 mental health agencies).

## Conclusions and directions for future research

The ChYMH was developed to support a coordinated and integrated (cross-sector) approach to the delivery of children’s mental health care services. Using data collected in Canada, it was found that the ChYMH demonstrated strong inter-consistency reliability, as well as good criterion validity, among a large sample of clinically referred children and youth. The present findings provide the first empirical data supporting the psychometric properties of the ChYMH, which has already had widespread uptake given its clinical utility. To extend these finding, additional research on the psychometric properties of the ChYMH with even larger samples of children and youth is warranted. In particular, assessments in different clinical care settings could allow for an examination of the psychometric properties of the ChYMH across diverse samples (e.g., inpatient vs outpatient vs primary care provider). Moreover, the ChYMH is also being translated for use in different languages, including French, thereby providing opportunities for cross-cultural comparisons. To further validate the assessment, it would also be worthwhile to include lab-based assessments of child behavior as criterion measures, to ensure the measures are assessing underlying mental health domains well across a variety of contexts. In addition, an important direction for future research will be to assess the test re-test reliability of the ChYMH using multiple assessment points, to examine the ChYMH’s effectiveness in assessing stability and change in mental health over time.

Given that the use of a standardized and comprehensive assessment could improve upon assessment information currently collected from non-standardized and non-integrated assessment instruments, research on the psychometric properties of the ChYMH is critically important. Our study provides strong support for the clinical utility of the instrument with clinically referred children and youth. Moving forward, the widespread usage of the ChYMH across various service providers could facilitate greater information sharing across service sectors (e.g., schools, mental health agencies, youth justice facilities, hospitals, daycares), ultimately to improve service system integration [47].

## Abbreviations

ADBS: Aggressive/Disruptive Behavior Scale
BCFPI: Brief Child and Family Phone Interview
CAFAS: Child and Adolescent Functional Assessment scale
CBCL: Child Behavior Checklist
ChYMH: Child and Youth Mental Health assessment
iNMH: interRAI Network of Excellence in Mental Health
ISD: interRAI Instrument and Survey Development Committee
RAI-MH: Resident Assessment Instrument - Mental Health
SSIS: Social Skills Improvement System
